# Reverse translational research of transient magnetic resonance imaging hyperintensity following intracranial stem cell therapy

**DOI:** 10.1186/s13287-025-04757-w

**Published:** 2025-11-18

**Authors:** Yi Qi, Masahito Kawabori, Yo Nakahara, Khin Khin Tha, Sumio Ohtsuki, Miki Fujimura

**Affiliations:** 1 Department of Neurosurgery, Faculty of Medicine, Hokkaido University, Kita 15, Nishi 7, Kita-ku, Sapporo, Hokkaido 060-8638 Japan; 2 Global Center for Biomedical Science and Engineering, Hokkaido University Faculty of Medicine, Sapporo, Japan; 3 Department of Pharmaceutical Microbiology, Faculty of Life Sciences, Kumamoto University, Kumamoto, Japan

**Keywords:** Transient FLAIR hyperintensity, Stroke, Intracranial, Stem cell therapy, Aquaporin-4

## Abstract

**Background:**

Neurological disorders, such as stroke and traumatic brain injury are the most significant global health challenges, leading to long-term physical and cognitive impairments. Cell transplantation therapy holds substantial promise for facilitating recovery, with numerous clinical trials currently underway. Intracerebral stem cell infusion offers the advantage of directly delivering a sufficient number of stem cells to the targeted area. Both our team and other researchers have observed a notable phenomenon following intracerebral stem cell therapy in clinical trials, wherein transient edema, as detected by Fluid Attenuated Inversion Recovery (FLAIR) magnetic resonance (MR) imaging, can be monitored between one and two weeks post-transplantation, with a subsequent resolution occurring approximately one month later. Notably, patient recovery appears to accelerate during the period of elevated FLAIR signals. However, the precise mechanisms underlying this distinctive phenomenon remain poorly understood. Therefore, this reverse translational research employs proteomics and histological analysis to investigate the mechanisms driving this phenomenon, thereby enhancing our understanding of stem cell therapy.

**Methods:**

Bone marrow-derived mesenchymal stem cells (BMSCs) were isolated from Sprague-Dawley (SD) rats, with passage 3 cells utilized for subsequent experiments. A total of one million cells, suspended in 10 µL of phosphate-buffered saline, were intracerebrally transplanted into the striatum of SD rats. Serial magnetic resonance imaging (FLAIR) scans were performed up to three weeks post-transplantation. Brain tissues were collected from the pre-signal (1 week post-transplantation), signal (2 weeks post-transplantation), and post-signal (3 weeks post-transplantation) groups for proteomic analysis, network analysis, and immunofluorescence imaging.

**Results:**

Consistent with clinical trials, transient FLAIR hyperintense signals were not detected until approximately two weeks post-intracranial stem cell therapy. These signals emerged around week two and diminished by week three. Proteomic analysis of brain specimens from the pre-signal and signal groups identified 231 differentially expressed proteins, which were primarily involved in vesicle-mediated transport, synaptic remodeling, and cellular communication. Glial fibrillary acidic protein (GFAP), Aquaporin-4 (AQP4), and Apolipoprotein E (APOE) were identified as key hub proteins. Immunofluorescence studies further confirmed that expression levels of GFAP, AQP4, and APOE increased around two weeks post-transplantation and significantly decreased by week three, coinciding with the resolution of the FLAIR signal.

**Conclusions:**

Our findings suggest that the transient FLAIR hyperintensity observed following intracerebral stem cell therapy is primarily attributed to transient glial cell activation, resulting in increased AQP4 expression and transient brain water influx. Additional mechanisms, including vesicle-mediated transport, secretion, synaptic activity, and lipid signaling, also contribute to the transient FLAIR hyperintensity signals and may play a role in the manifestation of clinical symptoms.

**Supplementary Information:**

The online version contains supplementary material available at 10.1186/s13287-025-04757-w.

## Introduction

Neurological disorders, including stroke and traumatic brain injury are the leading cause of long-term disability globally, which no effective treatment method has been established [1, 2]. In that circumstances, stem cell therapy has emerged as a promising treatment strategy for these diseases, and numerous clinical trials exploring stem cell treatment is being conducted worldwide [3–7]. Among them, intracerebral stem cell therapy possesses the advantage of delivering sufficient stem cell to the damaged area, compared with intra-venous transplantation, and our team has reported sufficient result of intracerebral stem cell therapy for ischemic stroke patients [8]. During this clinical trial, the authors have encountered a compelling phenomenon of transient brain edema at around the transplanted site observed by high signal in Fluid Attenuated Inversion Recovery (FLAIR) imaging using magnetic resonance (MR) imaging. The high signal starts to appears several days after cell transplantation and reaches peak between days 7 and 14, which subsequently diminishing over the following one to three months post-stem cell therapy. It is quite interesting that the patient recovery was accelerated during FLAIR high signaling period, including speech recovery as well as motor deficit. This temporal FLAIR high signal phenomenon has also been reported by other researchers [9–11], which positive correlation between the intensity of the hyperintense signals and clinical recovery has been reported. Despite these findings, the mechanisms underlying these transient hyperintense signals and its contribution to the functional recovery remain unclear. Evaluating and verifying the effects of stem cell therapy is particularly crucial and guide future research directions. Thus, this study investigates the molecular and cellular mechanisms underlying transient hyperintense signals observed in FLAIR MRI following intracranial stem cell therapy performed as reverse translational research.

## Methods

### Experimental design

**Fig. 1:**
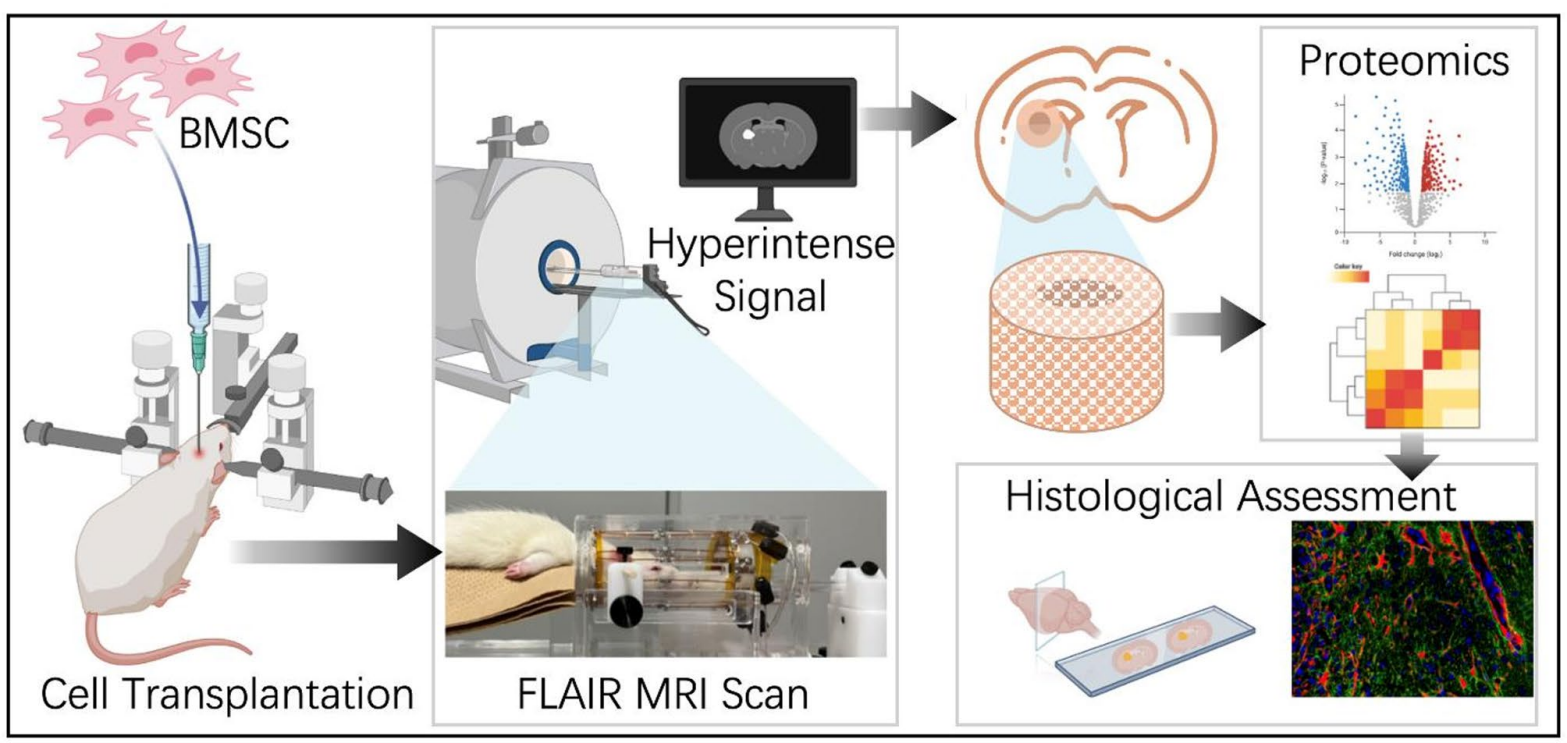
Experiment flow

This study aims to investigate the molecular mechanisms underlying the transient hyperintense signals observed in FLAIR MRI scans of patients undergoing intracranial stem cell therapy. To simulate this phenomenon, rat bone marrow mesenchymal stem cells (rBMSCs) were transplanted into the brains of Sprague-Dawley (SD) rats. Post-transplantation, in vivo FLAIR MRI scans were conducted at various time points over three consecutive weeks to monitor the occurrence and temporal dynamics of hyperintense signals. Rats were categorized into three groups based on FLAIR MRI findings: the Pre-signal group (rats without obviously FLAIR high signals), the Signal group (rats exhibiting FLAIR high signals) and the Post-signal group (rats with diminished FLAIR high signals) and each rat was considered a single experimental unit for the purposes of data collection and statistical analysis. Brain tissue samples from the transplantation sites were collected immediately after MRI scanning. Proteomic analysis was performed to compare the Pre-signal and the Signal groups, and subsequent differential protein expression analysis and network analysis were used to identify key proteins and signaling pathways involved. Proteins with high connectivity in the interaction network were prioritized for immunofluorescence (IF) validation. IF staining was employed to confirm the expression and spatial distribution of these candidate proteins in brain the transplantation sites. A simplified experiment flow is presented in Fig. 1. To minimize potential confounding factors, the order of stem cell infusion and MRI scan procedures was alternated across animals to avoid systematic bias. Cage positions within the animal facility were regularly rotated to reduce the influence of environmental variables such as light, temperature, and noise. In addition, the investigator performing the proteomic and MRI scan was independent.

### Rat BMSC harvest, culture and solution preparation

Bone marrow mesenchymal stem cells were isolated from the femurs of 6-7-week-old SD rats (CLEA Japan, Inc.), and sufficient cells were obtained for subsequent culture as previously reported [12–14]. Briefly, DMEM (Dulbecco’s Modified Eagle Medium, High Glucose) (Nacalai Tesque, Kyoto, Japan, Cat. No. 08458-16) containing heparin (10-fold dilution) was pre-prepared and supplemented with 50 ml of Fetal Bovine Serum (FBS)(Gibco, USA, Cat No. 10270, Gibco) and 10 mL of Penicillin-Streptomycin mixed solution (P/S) (Nacalai Tesque, Kyoto, Japan, Cat No. 26252-94). The rats were anesthetized with isoflurane gas, followed by euthanasia through cervical dislocation. The skin and muscles around the knee joint were quickly incised to expose the junction between the femur and tibia. Bone scissors were used to sever the femoral attachment at the knee side. The femur was then separated from the hip joint and completely removed. Both ends of the femur were cut to expose the bone marrow cavity, and any residual soft tissues were carefully trimmed in a container filled with the prepared medium. The femur was held with tweezers above a centrifuge tube, and the bone marrow cavity was gently flushed by slowly injecting the heparin-containing medium through a syringe into the top of the femur, allowing the bone marrow cells to flow out into the centrifuge tube below. The contralateral femur was processed using the same procedure to complete the collection of bone marrow cells. After centrifugation of the bone marrow cell suspension using KUBOTA S700FR benchtop centrifuge (KUBOTA Corporation, Japan) at 2 × 10³ rpm for 10 min, the supernatant was discarded. An appropriate volume of culture medium was added to resuspend the cells, followed by a second centrifugation at 2 × 10³ rpm for 5 min. The supernatant was then removed, and the cells were resuspended in the culture medium and seeded into BioLite™ T175 Flask (Thermo Scientific, REF 130191) using 25 ml of the heparin-free medium. The cultures were maintained in a CO₂ incubator, marking this passage as P1. The medium was replaced 1 to 2 days post-seeding, depending on cell attachment and proliferation, to purify rBMSCs. Subculture was performed on days 6–7 after initial seeding, following the procedure outlined below. Prior to detaching the cells, their morphology and growth status were examined under a microscope. The medium was aspirated, and the cells were rinsed with D-PBS (-) (1×) (Nacalai Tesque, Kyoto, Japan, Cat No. 14249-24). Subsequently, 4 mL of TrypLE Express (Gibco, USA, Cat No. 12604-021) was added and evenly distributed across the flask surface, followed by incubation at 37 °C for 5 min. Gentle tapping of the flask facilitated cell detachment, which was confirmed under a microscope. An equal volume (4 ml) of culture medium was added to neutralize the TrypLE, and the cell suspension was collected into Falcon tubes. The suspension was centrifuged using KUBOTA benchtop centrifuge at 1500 rpm for 5 min. The supernatant was discarded, and the pellet was gently loosened by tapping, followed by resuspension in 1 mL of culture medium. For cell counting and viability assessment, 2 µl of AO/PI staining solution was added to 18 µl of the cell suspension. After mixing, 10 µl of the mixture was loaded onto the Luna™ Cell Counting Slide (Logos Biosystems, Korea), and cell viability and concentration were assessed using the Luna™ FL Dual Fluorescence Cell Counter (Logos Biosystems, Korea). Cells were then seeded into new T175 flasks at a density of 2 × 10⁶ cells per flask. After confirmation under a microscope, the cultures were maintained at 37 °C in a CO₂ incubator. P3 passage rat BMSCs were used for intracranial stem cell infusion. For the preparation of the rBMSC suspension solution, P3 passage rBMSCs were detached, counted, and assessed for viability following the above-described method. Regarding the check of the MSC characteristics, this expansion method has previously shown to validate sufficient surface markers by flow cytometry analysis (FACS) in our lab [15–17], and microscopic analysis of spindle-shaped morphology and proliferative behavior was observed. The resulting cell suspension was transferred to 1.5 ml microcentrifuge tubes and centrifuged use Eppendorf Centrifuge 5417R (Eppendorf, Germany) at 2100 rpm for 5 min at 4 °C. After removing the supernatant, an appropriate volume of D-PBS (-) was added to adjust the cell concentration to 1 × 10⁶ cells/10 µL based on the live cell count. The prepared rBMSC suspension solution was kept on ice until used for intracranial stem cell infusion.

### Intracranial cell transplantation

In clinical, we observed that transient FLAIR hyperintense signals were concentrated around the cell transplantation site rather than around the ischemic lesion. Therefore, due to the compact anatomical structure of rat brain tissue, and to prevent interference from the infarcted tissue on MRI signals and proteomic analysis at the stem cell injection site, non-stroke model rats were used for intracranial stem cell infusion in this study. This decision was guided by the following clinic principle: the chosen transplantation site should reside within normal brain tissue, characterized by the absence of a FLAIR high signal [8]. A total of 59 rats were used in this study, including those used for preliminary experiments and experimental backups. Specifically, 21 rats were used for FLAIR MRI signal evaluation at different time points following stem cell infusion. For proteomic analysis, 4 animals were allocated to each group. For immunofluorescence staining, each group included 5 to 6 animals. The exact number of experimental units per group and per analysis is detailed in the corresponding figure legends and "Methods" sections. The sample size was determined based on preliminary experiments. This number was considered sufficient to detect biologically meaningful differences while minimizing animal use in accordance with ethical guidelines. The specific procedure for intracranial stem cell infusion in rats is described as follows: 9–10 weeks Male SD rats (CLEA Japan, Inc.) were used to perform the experimental intracranial stem cell therapy. Briefly, the rats were anesthesia by 2–5% isoflurane in 30% O_2_ and 70% N_2_O and the body temperature was maintained at between 36.5 and 37.5 °C throughout the procedures using the heat pad. The rat was positioned prone and stereotaxic procedure was performed using the Narishige SMM-100 stereotaxic manipulator (Narishige, Tokyo, Japan), and microinjection was performed using the Narishige IMS-20 injection control system (Narishige, Tokyo, Japan) to ensure precise control of injection speed and volume. After a midline scalp incision, to minimize potential interference from hemorrhage, a burr hole was carefully drilled under microscope, 3 millimeters lateral to the left of the bregma, down to the dura mater. A Hamilton 10 µL syringe (Hamilton Company, Reno, Nevada, USA) was used for automated injection. The needle was inserted 5 mm from the point where the needle tip touched the brain surface (beneath the skull), then advanced an additional 0.5 mm and retracted by 0.5 mm to create space for the fluid. The injection was performed at a rate of 0.041 µL/second, with a total injection volume of 10 µL. After completing the injection, the needle was left in place for 5 min to allow hemostasis and prevent backflow of the injected solution. The needle was then slowly withdrawn, and the scalp was sutured. Post-surgical monitoring was conducted regularly, and animals were returned to clean, warm cages after recovery. Procedures were designed to minimize invasiveness, and handling was kept gentle and consistent to reduce stress. Following successful rBMSCs transplantation, a series of in vivo FLAIR MRI scans were performed over consecutive days to evaluate the presence and progression of hyperintense signals at the transplantation site in the post-transplantation model. Humane endpoints were established prior to the study. Animals were monitored daily for signs of pain or distress, including weight loss (>20%), decreased mobility, abnormal posture, self-mutilation. Animals were humanely euthanized under deep anesthesia induced with 5% isoflurane in a mixture of 30% O₂ and 70% N₂O, followed by cervical dislocation in accordance with institutional and ethical guidelines. Inclusion and exclusion criteria were established a priori. Animals were included in the study if they met the following criteria: successful stem cell infusion without surgical complications, and completion of the designated experimental procedures. Animals were excluded if they exhibited signs of severe distress, unexpected mortality, or technical failure (e.g., unsuccessful MRI scanning or tissue collection).

### T2-FLAIR MRI scan

All MRI scans were performed in vivo on anesthetized rats. Anesthesia was induced and maintained using 4% isoflurane delivered in a gas mixture of 30% O₂ and 70% N₂O. The scans were conducted using a 3.0 Tesla MAGNETOM Prisma scanner (Siemens Healthineers, Erlangen, Germany) equipped with an eight-channel birdcage coil for rodent models (Takashima Seisakusho Co. Ltd., Japan]). T2-FLAIR (TE: 125 ms, TR: 5000 ms, voxel size = 1.5 mm) and T2-3D FLAIR imaging (TE: 410 ms, TR: 4000 ms, voxel size = 0.4 mm) was performed to detect local brain changes at the transplantation site on days 1, 3, 5, 7, 9, 10, 12, 14, 16, 18, and 20 post-transplantation. Both 2D FLAIR and 3D FLAIR are used to evaluate the signal of the transplant site. 3D FLAIR is only used for observation and position evaluation. All signal-intensity quantitative analyses use 2D FLAIR images. Unsuccessful MRI scanning was not included (*n* = 4) because severe artifacts due to animal breath activity or rat death when MRI scanning.

For quantitative analysis of the FLAIR signal at the transplantation site, the maximum signal intensity was measured using a region of interest (ROI) placed precisely at the stem cell infusion site. Consistency in ROI size (circular, w = 140, h = 140, number of pixels: 15380) and placement was ensured across all images. A control ROI of the same size was placed in an air region outside the rat, maintaining the same position across all images to account for the background signal. The relative maximum signal intensity ratio between the transplantation site ROI and the control ROI was calculated using the 95th percentile of the maximum signal intensity. This approach was chosen to reduce the influence of potential outliers or noise artifacts. MRI image analysis and quantification were performed using ImageJ software (version 1.53k, National Institutes of Health, USA, https://imagej.nih.gov/ij/).

### Brain tissue samples collection and processing

Following FLAIR MRI scans, rats were sacrificed immediately after scanning at two specific time points: when no hyperintense signals were observed (day 7 post-transplantation) (*n* = 4) and when hyperintense signals were detected (approximately 2 weeks post-transplantation) (*n* = 4). Experimental groups were assigned based on pre-matched according to FLAIR MRI signals. Perfusion was performed using 200 mL of saline, after which the brains were harvested and instantly frozen in liquid nitrogen before being stored at -80 °C.

To maximize the accuracy of the proteomic results, brain tissue was collected with a coronal thickness of 4 mm, centered at the stem cell infusion site. And a 4 mm circular blade was used to precisely isolate the transplantation site, yielding approximately 25–30 mg of tissue. The corresponding contralateral region of the brain, without stem cell infusion, was collected as the quality control group. Brain tissue samples were homogenized in 1 ml of homogenizing buffer (101 mM NaCl, 4.6 mM KCl, 2.5 mM CaCl₂, 1.2 mM KH₂PO₄, 1.2 mM MgSO₄, and 15 mM HEPES, pH 7.4) with 3 steel beads using the ShakeMan3 homogenizer (BMS-SMN03, BMS, Japan) at a speed of 310 rpm for 30 s. The homogenized tissue mixture was aliquoted into 1.5 ml protein storage tubes, with 500 µl per tube. Subsequently, 500 µl of 2X lysis buffer was added to each tube. The 2X lysis buffer consisted of 24 mM Sodium Deoxycholate (Wako Pure Chemical Industries, Japan, Cat# 192–08312), 24 mM Sodium N-dodecanoylsarcosinate (Wako Pure Chemical Industries, Japan, Cat# 198-14745), Tris-buffered saline (pH 8.0, powder; Sigma-Aldrich, Cat#T6664), and cOmplete™ Mini Protease Inhibitor Cocktail (Roche, Cat#11836153001) at a concentration of 1 tablet per 5 mL of lysis buffer. The samples were dissolved on ice using Bransonic 8510 ultrasonication (Yamato/Branson Ultrasonics Corporation, USA) for 1 min, followed by incubation at 37 °C for 30 min. After incubation, the samples were centrifuged using Eppendorf Centrifuge 5417R at 15,000 × g for 15 min at 4 °C. The resulting supernatant was carefully collected into 2 ml storage tubes. A portion of the supernatant was reserved for protein quantification using the Pierce™ BCA Protein Assay Kit (Thermo Scientific, USA, Cat#23227,) according to the manufacturer’s instructions. The remaining supernatant was aliquoted and storage at -80 °C for subsequent proteomic analysis.

### Data independent acquisition (DIA)‑based quantitative proteomics

Brain tissue from the transplantation site was collected at two specific time points for quantitative proteomics: the Signal group defined when high signals appeared in FLAIR MRI (approximately around 2 weeks post-transplantation) and the Pre-signal group defined when no significant FLAIR signals were observed (7 days post-transplantation). Contralateral brain tissue from the right side served as the control side tissue. Samples were digested using the SP3 method as previously described [18, 19]. A TripleTOF 7600 mass spectrometer (Sciex, Framingham, MA, USA) coupled with the Dionex Ultimate 3000 RSLCnano System (Dionex, Sunnyvale, CA, USA) was used for DIA-MS. The peptides and proteins were identified and quantified using DIA-NN 1.8.1 with UniProt rat reference proteome data and filtering at a false discovery rate of < 1%. Differential expression protein analysis was performed using RNAseqChef (https://imeg-ku.shinyapps.io/RNAseqChef_imeg/) [20]. Pairwise differential expression analysis between the Pre-signal group and the Signal group was conducted using the limma package for normalized count data, as implemented in RNAseqChef. Given the exploratory nature of this study, proteins with false discovery rate (FDR) less than 0.1 were considered as significantly differentially expressed, if those proteins also had a fold change of lower/higher than -1.2/1.2, they were defined as being down-/upregulated, respectively. Network and Gene Ontology (GO) analysis of the differentially expressed proteins was conducted using STRING version 12.0 (https://string-db.org/) [21]. Protein degree analysis was performed using Cytoscape software (Version 3.10.3) to identify key proteins within the network based on their connectivity and influence [22]. 

### Validation of selected proteins by immunofluorescence (IF)

Following differential proteomic analysis and protein degree analysis between the Pre-signal group and the hyperintense-signal group, three proteins—glial fibrillary acidic protein (GFAP), aquaporin-4 (AQP4), and apolipoprotein E (APOE)—with increased expression in the Signal group were selected for validation using IF staining. Spatial distribution and quantitative analysis were performed. Rats were sacrificed at three specific time points: when not obviously hyperintense signals were observed (day 7 post-transplantation), when hyperintense signals were detected (approximately 2 weeks post-transplantation), and when hyperintense signals had disappeared (3 weeks post-transplantation). During sacrifice, the rats were deeply anesthetized and transcardially perfused with sodium chloride injection solution, followed by 10% paraformaldehyde (PFA). The brains were extracted, fixed in 10% PFA for 24 h, and embedded in paraffin as previously described [12, 23]. Coronal sections (4 μm) were prepared using the Leica RM2125 manual rotary microtome (Leica Microsystems, Wetzlar, Germany) centered on the stem cell infusion site. IF staining was performed as previously described protocols [24]. Briefly, tissue sections were deparaffinized with xylene, and antigen retrieval was performed using Citrate buffer (pH 6.0) at 170 °C for 3 min followed by a 30-minute blocking to decrease non-specific binding. For co-staining of GFAP and AQP4, primary antibodies—mouse anti-GFAP antibody (BD, Cat# 610565, 1:400) and rabbit anti-AQP4 antibody (Proteintech, Cat# 16473-1-AP, 1:200)—were incubated at room temperature for 60 min. Secondary antibodies—Alexa Fluor™ 594 goat anti-mouse IgG (H + L) antibody (Invitrogen, Thermo Fisher Scientific, USA, Cat#A-11005, 1:1000) and Alexa Fluor™ 488 goat anti-rabbit IgG (H + L) antibody (Invitrogen, Thermo Fisher Scientific, USA, Cat#A-11008, 1:1000)—were incubated at room temperature for 60 min. For APOE staining, the primary antibody used was rabbit anti-APOE antibody (Abcam, Cat# ab183597, 1:500), incubated at room temperature for 60 min. The secondary antibody was Alexa Fluor™ 594 goat anti-rabbit IgG (H + L) antibody (Invitrogen, Thermo Fisher Scientific, USA, Cat#A-11012, 1:1000), incubated at room temperature for 60 min. Samples were mounted using ProLong™ Diamond Antifade Mountant with DAPI (Invitrogen, Thermo Fisher Scientific, USA, Cat#P36962) to preserve fluorescence signals and counterstain nuclei.

Images were acquired using the KEYENCE BZ-X710 microscope (Keyence, Osaka, Japan), focusing on regions adjacent to the peri-transplantation site under consistent conditions. A total of five random, non-overlapping fields were selected for each section, and positive signal area and fluorescence intensity were quantified under consistent conditions using automated cell counting software BZ-X Analyzer (Hybrid Cell Count, Keyence, Osaka, Japan).

### Statistics

All numerical data are expressed as mean ± standard error of the mean (SEM) values. Statistical significance of the differences between the means was determined using the unpaired two-tailed Student’s t-test and Welch’s t-test for two groups and one-way analysis of variance (ANOVA) followed by Tukey’s test for more than two groups. All statistics analyses were performed using GraphPad Prism 9 (GraphPad Software, San Diego, CA, USA) and Microsoft Excel (Microsoft, Redmond, WA, USA).

## Results

### Appearance of transient FLAIR high signals in the rat intracranial stem cell therapy model

**Fig. 2:**
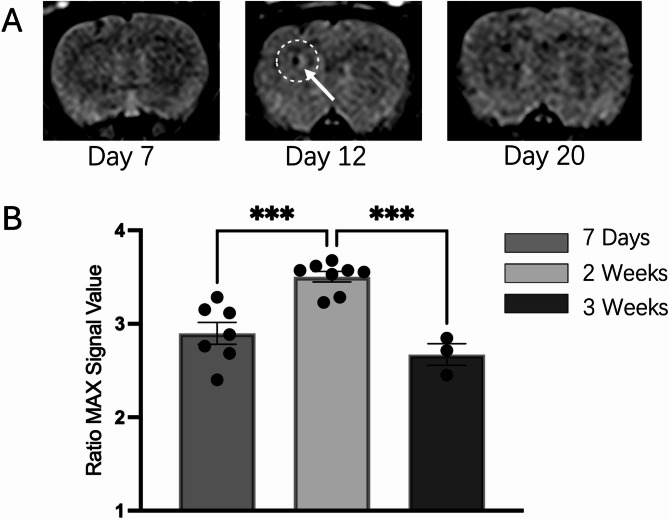
FLAIR MRI Scan and signal quantify analysis. **A** Representative cases in Pre-signal, Signal (white arrow) and Post-signal; **B** Dynamic Quantitative Analysis of FLAIR Signals: The 95th percentile of the relative maximum signal intensity was used to quantify FLAIR signals across different time points. A significant increase in signal intensity was observed around 2 weeks post-transplantation (*n* = 8) compares with 7 days post-transplantation (*n* = 7), followed by a decline by the third week (*n* = 3). *P < 0.05, ** *P* < 0.01, *** *P* < 0.001, **** *P* < 0.0001

In this study, in vivo FLAIR MRI scans were performed at various time points following intracranial stem cell infusion in rats. We observed that no significant hyperintense signals were detected at day 7 post-transplantation, followed by transient hyperintense signals approximately two weeks post-transplantation. Furthermore, the signals diminished approximately three weeks after transplantation (Fig. 2A). To further quantify the FLAIR signals, we compared the 95th percentile maximum relative signal intensity values at the transplantation site. The signal intensity was significantly higher in approximately two weeks post-transplantation compared to day 7 post-transplantation (*P* = 0.0005). Moreover, the signal intensity significantly decreased by the third week post-transplantation compared to the second week (*P* = 0.0003) (Fig. 2B). These findings confirm that the transient hyperintense signal reaches its peak around the second week and diminishes by the third week following stem cell infusion in our rat model. This temporal pattern is consistent with clinical observations, indicating that the rat intracranial infusion model successfully replicates the transient hyperintense signals seen in patients undergoing intracranial stem cell therapy.

### Changes in protein expression of the FLAIR high signal tissue

**Fig. 3:**
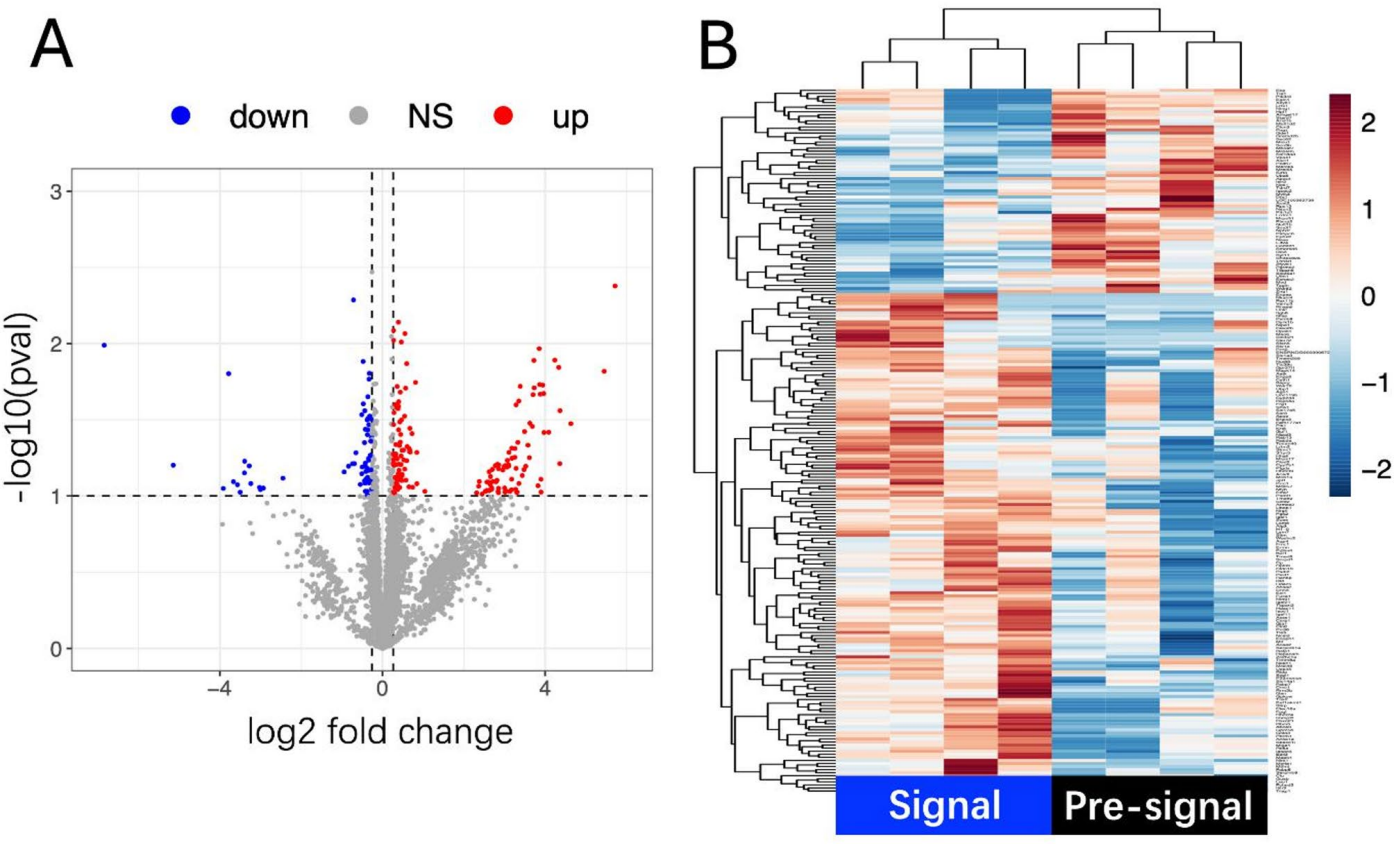
Summary of Proteomics Results. **A** The volcano plot highlights differentially expressed proteins between the Signal and Pre-signal groups, with 164 proteins upregulated in the Signal group; **B** The heatmap demonstrates distinct clustering of upregulated proteins in the Signal group compared to the Pre-signal group. (*n* = 4 each group)

**Fig. 4:**
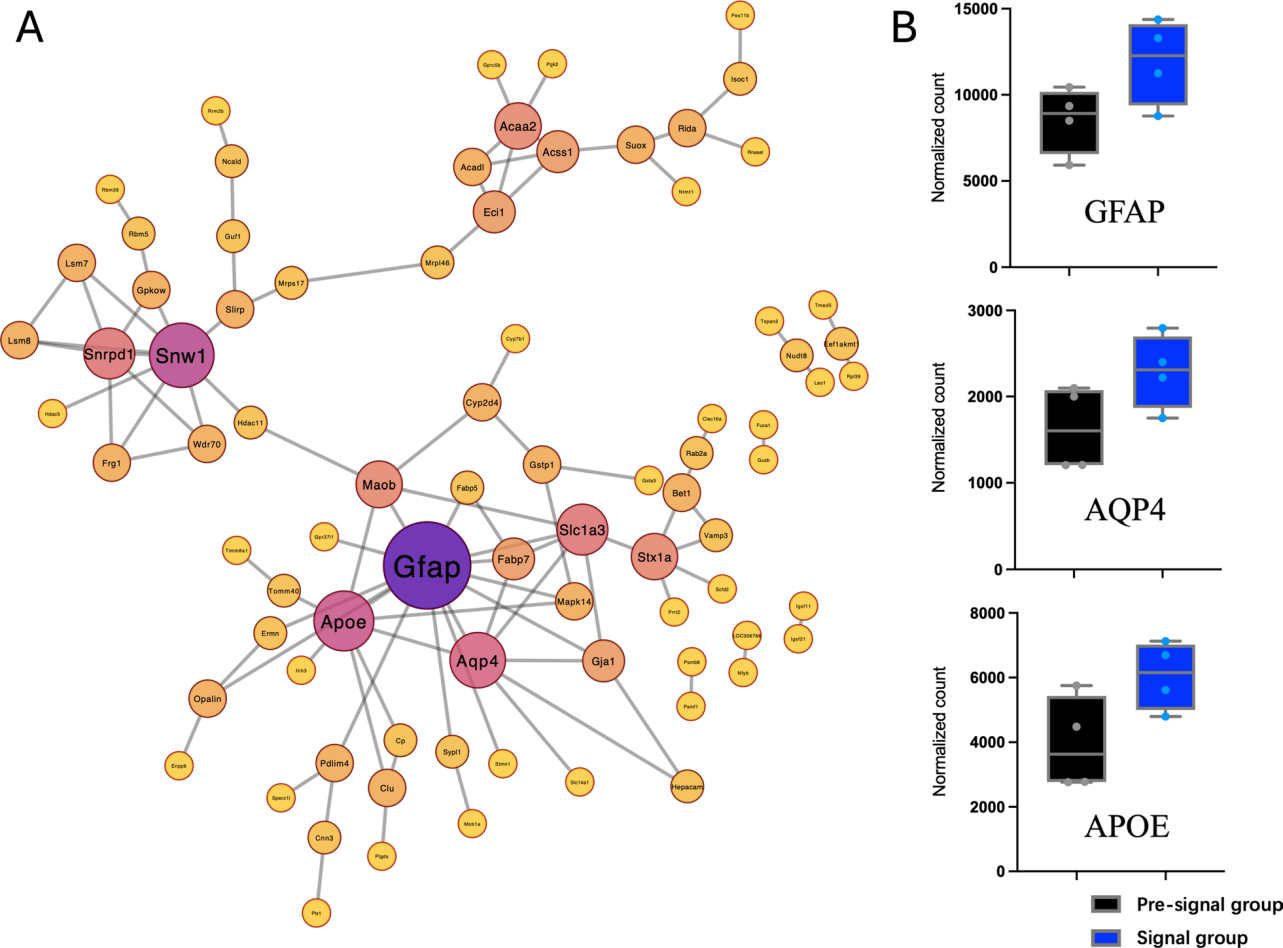
PPI Network and hub proteins. **A** A protein-protein interaction (PPI) network was constructed for upregulated proteins in the Signal group, revealing three distinct clusters. Degree analysis identified key hub proteins within the largest cluster, including GFAP, AQP4, and APOE; **B** Quantitative proteomics results of the hub proteins GFAP, AQP4, and APOE in the Signal group compared to the Pre-signal group

**Fig. 5:**
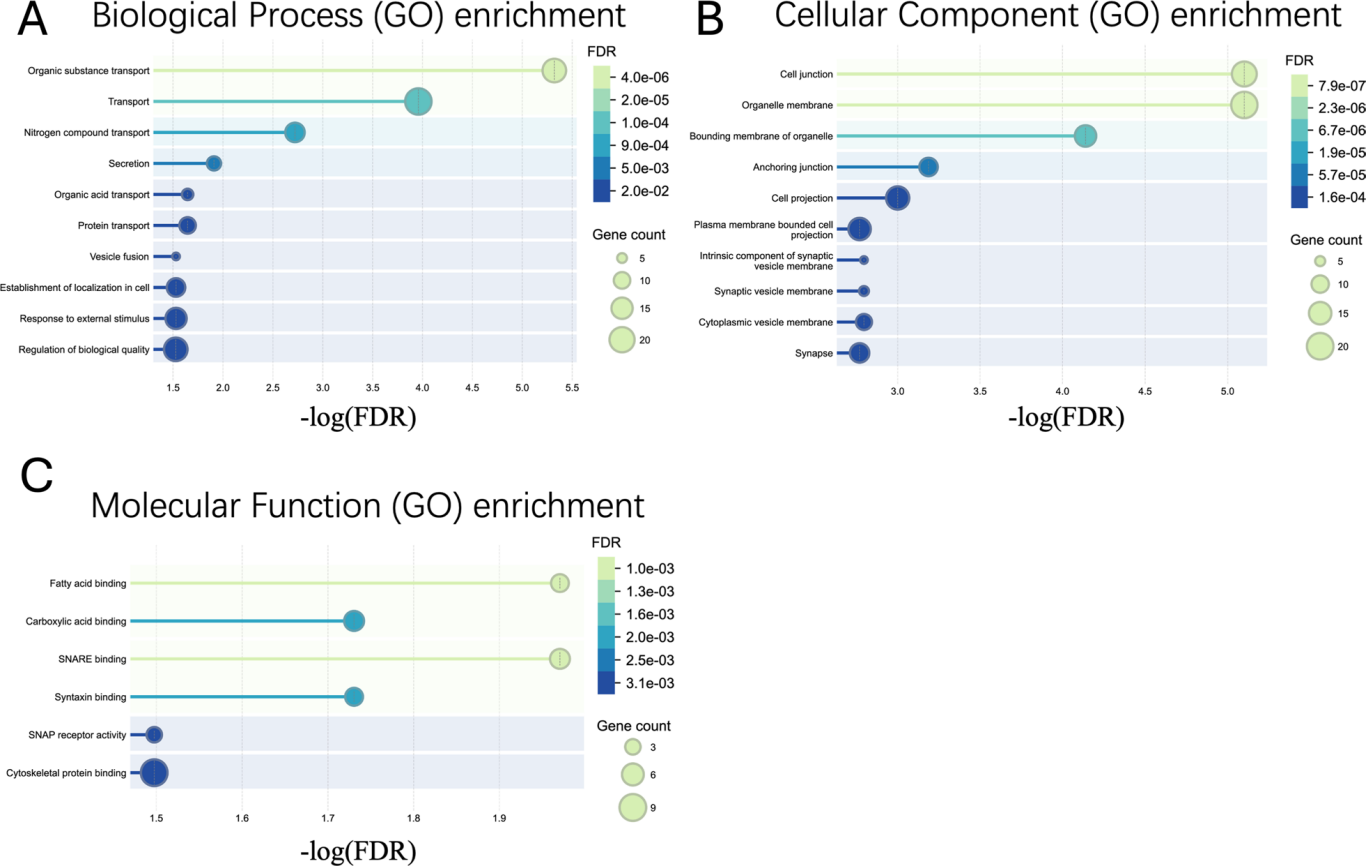
Gene Ontology (GO) Analysis. **A** Biological process (GO) enrichment; **B** Cellular Component (GO) enrichment; **C** Molecular Function (GO) enrichment

Proteomic analysis of brain tissue samples from the transplantation sites identified 6,548 proteins, among which 231 were differentially expressed between the Signal and Pre-signal groups (*Fold Change ≥* 1.2, *FDR ≤* 0.1). Detailed proteomics data are available on supplementary file (Supple 1 and 2). Of these, 164 proteins were upregulated and 67 downregulated in the Signal group (Fig. 3). Protein-protein interaction analysis of the 164 upregulated proteins using STRING identified three cluster and the largest cluster consisting of 39 interconnected proteins (Fig. 4A). GO enrichment analysis of the largest cluster revealed significant involvement in biological processes such as Organic substance transport (GO:0071702; *FDR* = 4.78E-06), Transport (GO: 0006810; *FDR* = 0.00011), secretion (GO:0046903; *FDR* = 0.0123) and Vesicle fusion (GO:0006906; *FDR* = 0.0295), Establishment of localization in cell (GO:0051649; *FDR* = 0.0295) (Fig. 5A). These processes suggest active molecular trafficking and communication within the host brain following stem cell infusion. Cellular component analysis revealed significant associations with Cell junctions (GO:0030054; *FDR* = 7.92E-06), Organelle membranes (GO:0031090; *FDR* = 7.92E-06), and Synaptic vesicle membranes (GO:0030672; *FDR* = 0.0016) (Fig. 5B), indicating that the transient FLAIR signals stage linked to synaptic remodeling and cellular communication. In terms of molecular function, enriched terms included Fatty acid binding (GO:0005504; *FDR* = 0.0107), SNARE binding (GO:0000149; *FDR* = 0.0107),and Cytoskeletal protein binding (GO:0008092; *FDR* = 0.0318) and so on (Fig. 5C).

These findings highlight the critical role of vesicle-mediated transport, secretion, and synaptic activity in the transient hyperintense signals observed in FLAIR MRI. The upregulation of proteins involved in SNARE binding and vesicle dynamics suggests that these processes are central to the interaction between transplanted cells and the host brain tissue. Furthermore, the enrichment of cellular components such as synaptic vesicle membranes and cell junctions underscores the significance of synaptic remodeling and cellular communication in this phenomenon stage.

### GFAP, AQP4 and APOE are central hub proteins

Through protein degree analysis of the PPI network, key hub proteins were identified based on their connectivity and centrality. This analysis highlighted GFAP, AQP4, and APOE as central proteins within the largest cluster. In the quantitative proteomics results (Fig. 4B), the mean GFAP expression was 8,553.3 ± 2,022.8 in the Pre-signal group and 11,922.4 ± 2,300.4 in the Signal group, representing a significant increase (fold change ~ 1.39). This result highlights the role of astrocytic activation in the Signal group. The mean AQP4 expression was 1,628.53 ± 466.8 in the Pre-signal group and 2,292.55 ± 444.6 in the Signal group, representing a significant increase (fold change ~ 1.41). This increase in AQP4 expression in the Signal group suggests enhanced water transport and osmotic regulation. The mean APOE expression was 3,944.33 ± 1,340.6 in the Pre-signal group and 6,058.19 ± 1,033.1 in the Signal group, representing a notable increase (fold change ~ 1.54). This upregulation in the Signal group highlights the involvement of APOE in lipid transport, synaptic remodeling, and neuroinflammation regulate.

### Verification for GFAP, AQP4 and APOE

From the largest cluster identified through proteomic analysis, three proteins—GFAP, AQP4 and APOE—were selected for validation due to their high degree of connectivity in network analysis and their established roles in glial activity, water transport, and lipid metabolism, respectively. IF staining was performed on the Pre-signal group, Signal group and Post-signal group to confirm their localization and relative expression levels at the stem cell infusion site.

**Fig. 6:**
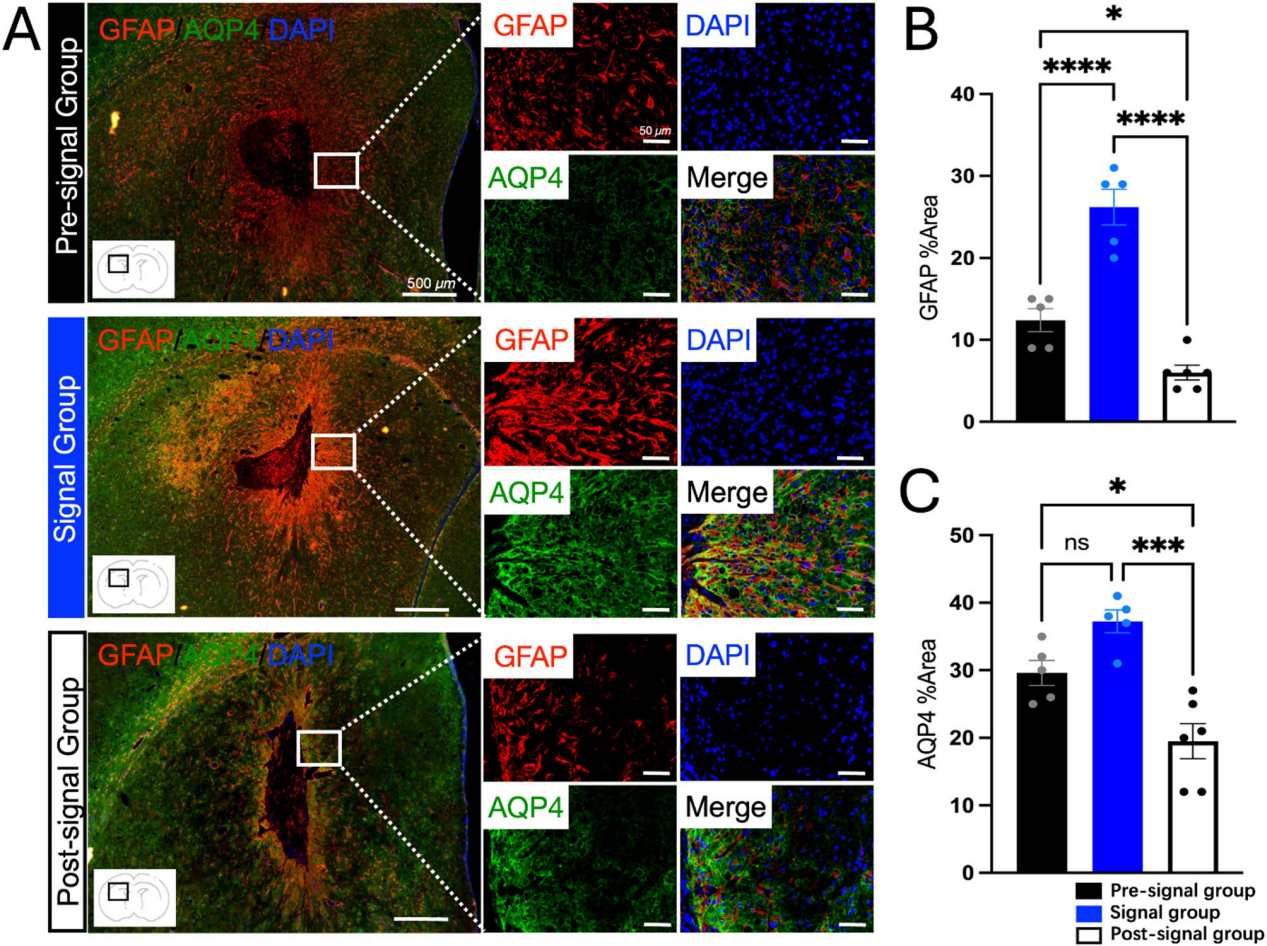
Verification of GFAP and AQP4 by Co-staining Immunofluorescence. **A** Representative Images of GFAP and AQP4 Co-staining: IF staining of GFAP and AQP4 in the Pre-signal, Signal groups and Post-signal group. Strong positive staining for both proteins was observed around the stem cell infusion site in the Signal group and a notable decrease in the expression intensity of both proteins was observed in the Post-signal group compared to the Pre-signal group. Scale bar = 500 μm in insets, 50 μm in higher magnification images; **B** Quantification of GFAP Staining: The positive area of GFAP staining were significantly increased in the Signal group compared to the Pre-signal group (*P* < 0.0001) and significantly decreased in the Post-signal group compared to the Signal group (*P* < 0.0001); **C** Quantification of AQP4 Staining: The positive area of AQP4 staining were increased trend in the Signal group compared to the Pre-signal group (*P* = 0.0776) and significantly decreased in the Post-signal group compared to the Signal group (*P* = 0.0002). (*n* = 5 ~ 6 each group) *P < 0.05, ** *P* < 0.01, *** *P* < 0.001, **** *P* < 0.0001

In the co-staining of GFAP and AQP4, both proteins were predominantly localized at the edges of the cell transplantation site (Fig. 6A). Compared to the Pre-signal group, the Signal group exhibited significantly stronger fluorescence signals in GFAP and increased trend in AQP4. Quantitative analysis of the positive area revealed that GFAP increased by 2.06-fold in the Signal group compared to the Pre-signal group (*P* < 0.0001) (Fig. 6B), and AQP4 increased by 1.26-fold (*P* = 0.0776) (Fig. 6C).

**Fig. 7:**
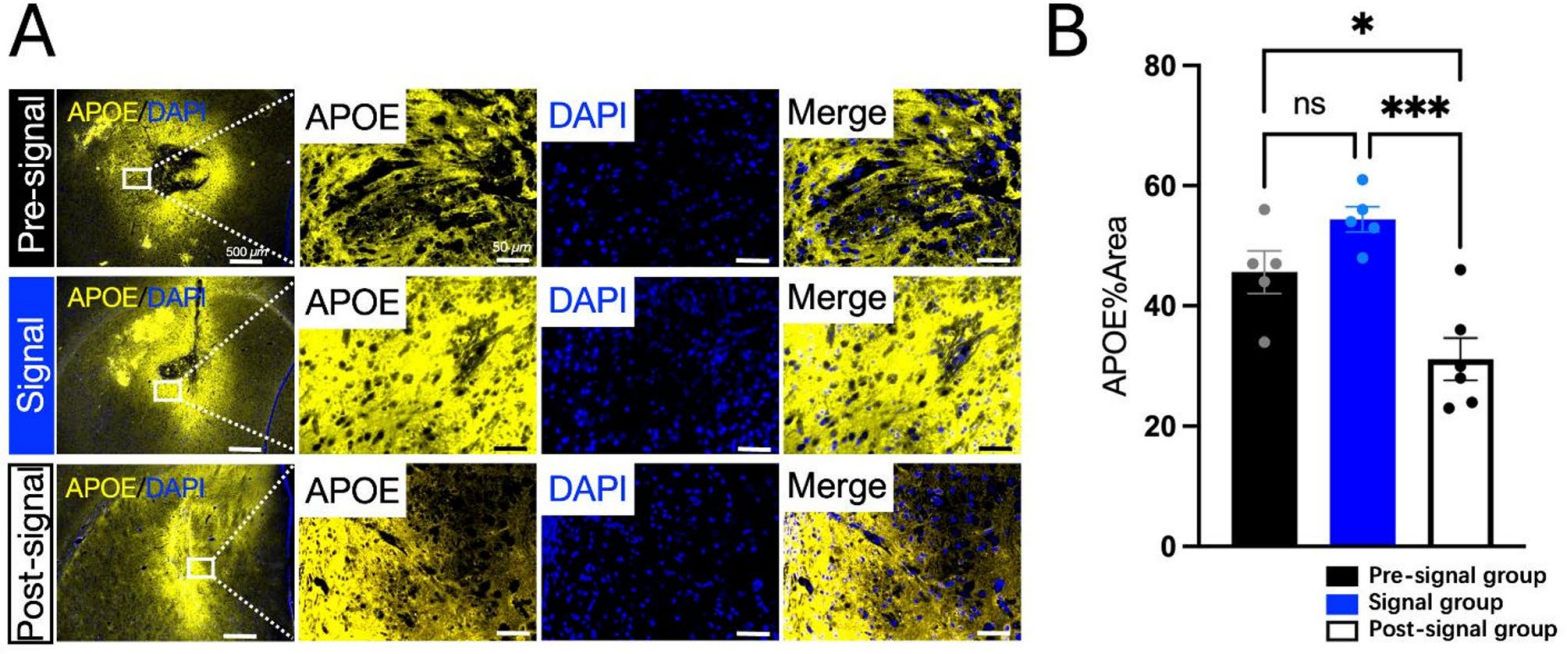
Verification of APOE by Immunofluorescence. **A** Representative images of APOE Staining: IF staining of APOE in the Pre-signal, Signal groups and Post-signal group. Relatively strong positive staining for APOE was observed around the stem cell infusion site in the Signal group. Scale bar = 500 μm in insets, 50 μm in higher magnification images; **B** Quantification of APOE Staining: The positive area of APOE staining showed a trend toward an increase in the Signal group compared to the Pre-signal group (*P* = 0.1833) and significantly decreased in the Post-signal group compared to the Signal group (*P* = 0.0005). (*n* = 5 ~ 6 each group) *P < 0.05, ** *P* < 0.01, *** *P* < 0.001, **** *P* < 0.0001

For APOE, the protein was also predominantly localized around the stem cell infusion site (Fig. 7A). Quantitative analysis indicated a 1.19-fold increase in the positive area for APOE in the Signal group compared to the Pre-signal group (*P* = 0.1833) (Fig. 7B).

These findings are consistent with the proteomic analysis results (Fig. 4B), which identified GFAP as significantly upregulated in the Signal group, and AQP4 and APOE as showing an upregulated trend.

### Downregulation of GFAP, AQP4 and APOE corresponds to the FLAIR high signals decline by the third week

Following the series of FLAIR MRI scans, the FLAIR signal was observed to peak approximately two weeks after intracranial stem cell infusion and decline by the third week around. To investigate the temporal dynamics of GFAP, AQP4 and APOE expression, IF staining was performed on brain tissues from rats three weeks post-transplantation (Post-signal group).

The results demonstrated that, compared to the peak FLAIR signal observed at approximately two weeks post-transplantation, the fluorescence signals of GFAP and AQP4 surrounding the stem cell infusion site was significantly reduced by the third week (Fig. 6A). Quantitative analysis of the positive area revealed that GFAP significantly decreased by 4.13-fold in the Post-signal group compared to the Signal group (*P* < 0.0001), and AQP4 significantly decreased by 1.88-fold (*P* = 0.0002) (Fig. 6B, C). For APOE, the protein was also significantly reduced by the third week (Fig. 7A). Quantitative analysis indicated a significantly decreased by 1.74-fold in the Post-signal group compared to the Signal group (*P* = 0.0005) (Fig. 7B). These findings reveal a temporal progression of AQP4 expression following intracranial stem cell infusion: mild upregulation at day 7 (no significant FLAIR signal), followed by widespread and diffuse significantly upregulation around the transplantation site at two weeks (coinciding with the peak FLAIR high signal), and finally almost only localized at the transplantation site boundary with significantly decreased area and intensity by the third week (coinciding with the decline in FLAIR signals). This temporal and spatial pattern of AQP4 expression suggests its critical involvement in the dynamic regulation of osmotic balance in response to stem cell therapy.

## Discussion

This research using a rat model, successfully replicated the temporal pattern of transient FLAIR hyperintense signals reported in clinical trials. In our rat model, the FLAIR hyperintense signals reaching a peak around two weeks post-transplantation and diminishing by the third week. Through the integration of proteomics techniques and immunofluorescence validation, we were able to demonstrated that transient glial activation followed by AQP4 activation were the main mechanisms of transient edema. Other factors including molecular trafficking and communication within the host brain following stem cell were considered to contribute to the functional recovery.

In the Signal group, the proteomic analysis revealed 231 differentially expressed proteins compare with the Pre-signal group, with 164 upregulated proteins in the Signal group. GO enrichment and network analyses identified upregulated pathways related to cell junctions, vesicle-mediated transport, secretion, synaptic vesicle membranes, synaptic activity and cytoskeletal remodeling. These findings suggest that the functional roles of transplanted stem cells are highly activated during this period, potentially contributing to neurovascular remodeling and functional recovery. The establishment of communication between transplanted stem cells and the host, as well as intercellular communication, is one of the key mechanisms through which their functions are exerted [25]. Stem cells can regulate the neurovascular microenvironment through autocrine or paracrine activities, including the release of cytokines, growth factors, and extracellular vesicles, thereby promoting tissue repair and regeneration [26, 27]. Moreover, stem cells can influence gene expression in recipient cells under both physiological and pathophysiological conditions by delivering cargo such as synaptic proteins, non-coding RNAs, DNA, and lipids [28–30]. 

Brain edema is a primary cause of FLAIR signal enhancement, resulting from excess fluid accumulation in brain tissue. The key types of edemas include vasogenic edema, caused by blood-brain barrier disruption, and cytotoxic edema, resulting from cellular swelling during ischemia or hypoxia [31, 32]. Other types include interstitial edema, linked to hydrocephalus; osmotic edema, due to plasma-brain osmolality imbalances like hyponatremia; [33] inflammatory edema, from autoimmune or infectious conditions; and tumor-associated edema, combining vasogenic effects and mechanical compression [34]. In our study, proteomic analysis combined with PPI network degree analysis identified AQP4 is a key protein in the Signal group. AQP4, a key water channel protein, plays a role in osmotic regulation and is implicated in brain edema and cellular fluid homeostasis [33, 35, 36]. In our study, we observed that during the signal phase, the expression of AQP4 around the stem cell infusion site was significantly elevated. Increased and disorganized AQP4 expression disrupt water homeostasis and leading to localized edema. In the study by Philip Kitchen published in Cell [33], a direct mechanistic relationship between AQP4 and central nervous system (CNS) edema was demonstrated. The research showed that AQP4 translocation to the astrocyte cell surface increases water flux, contributing to edema formation. Therefore, the transient overexpression and distribution of AQP4 observed in the signal group is key a contributing factor to the development of transient edema following intracranial stem cell therapy.

Another key protein identified in this study through proteomic and network degree analysis was GFAP, a marker of astrocytic activation. AQP4 is expressed in astrocytes and mediates water flux across the blood-brain barrier. The proteomic analysis and immunofluorescence staining results in this study demonstrated that the expression of AQP4 and GFAP exhibited a dynamic pattern consistent with the FLAIR signal, showing an initial increase followed by a subsequent decrease. GFAP is associated with gliosis and structural support in the central nervous system. We observed high expression of GFAP in the Signal group. In the study by Barbara Klein et al., hMSC-SB623 cell treatment for focal ischemic stroke in rats show that hMSC-SB623 cell treatment significantly enhanced GFAP expression in the ipsilesional cortex and corpus callosum, as well as in the peri-stroke region and increases proliferation of GFAP + astrocytes. And which suggests that GFAP upregulation is linked to the proliferative response of astrocytes, contributing to neural plasticity and functional recovery after stroke [37]. Astrocytes play a dual role in the CNS: they support recovery after injury by providing neurotrophic factors such as BDNF, and they regulate neuronal signaling through synaptic modulation and neurotransmitter recycling [38–42]. 

In addition, in this research, APOE was identified as another key hub protein during the signal phase, and which exhibited an upward trend in the Signal group. APOE is well known for its roles in lipid transport and neuroinflammation and is closely associated with synaptic remodeling and neural repair. Elevated levels of APOE may suppress inflammation and facilitate lipid delivery to neurons, thereby enhancing neural resilience and promoting recovery [43]. 

The identification of GFAP, AQP4, and APOE as central hub proteins highlights their potential as biomarkers for monitoring brain responses following stem cell therapy. Furthermore, the area or intensity of transient FLAIR signals may reflect stem cell activity, functionality, and their ability to establish connections with the host. Targeting these proteins and the pathways they regulate could offer therapeutic opportunities to modulate the host brain’s response, thereby enhancing the safety and efficacy of stem cell therapy in future.

These findings shed light on the molecular mechanisms underlying the transient hyperintense signals observed in FLAIR MRI after intercranial stem cell therapy, suggesting that they are primarily driven by astrocytic activation, osmotic regulation, and synaptic remodeling rather than pathological damage. The benign nature of these signals, as demonstrated by their resolution and lack of association with unfavorable outcomes, highlights their potential as markers of post-transplantation tissue responses.

While this study provides valuable insights, several limitations should be acknowledged. First, the proteomic analysis was restricted to two time points; a more extensive temporal analysis could provide a deeper understanding of the dynamic changes underlying the FLAIR signals. Second, this study focused on a single type of stem cell, rat derived MSC for infusion, leaving it unclear whether different stem cell types, transplantation dosages, or transplantation sites might influence the occurrence of transient hyperintense signals. Future research should explore these variables to better understand the mechanisms involved. Furthermore, future studies should investigate the clinical relevance of these findings by examining whether similar molecular changes occur in human patients undergoing stem cell therapy. Such research could bridge the gap between experimental models and clinical practice, paving the way for improved stem cell therapeutic strategies.

## Conclusion

This study first elucidates the molecular and cellular basis of transient hyperintense signals observed in FLAIR MRI following intracranial stem cell infusion. The findings highlight the roles of vesicle-mediated transport, gliosis, osmotic regulation, and lipid signaling in this phenomenon and identify GFAP, AQP4, and APOE as key players. These results not only advance our understanding of the host brain’s response to stem cell therapy but also provide potential biomarkers and therapeutic targets for improving cell transplantation outcomes.

## Supplementary Information

Below is the link to the electronic supplementary material.

Supplementary Material 1. Supplementary table 1: Groups used in protemics.

Supplementary Material 2. Supplementary table 2. Raw data of proteomics.

## Data Availability

All additional files are included in the manuscript. The data that support the findings of this study are available from the corresponding author, [MK], upon reasonable request.
